# Long‐term retention in pre‐exposure prophylaxis care among men who have sex with men and transgender women in the United States

**DOI:** 10.1002/jia2.25385

**Published:** 2019-08-18

**Authors:** Philip A Chan, Rupa R Patel, Leandro Mena, Brandon DL Marshall, Jennifer Rose, Cassandra Sutten Coats, Madeline C Montgomery, Jun Tao, Collette Sosnowy, Kenneth H Mayer, Amy Nunn

**Affiliations:** ^1^ Department of Medicine Brown University Providence RI USA; ^2^ Department of Behavioral and Social Sciences Brown University School of Public Health Providence RI USA; ^3^ Department of Medicine Washington University in St. Louis St. Louis MO USA; ^4^ Department of Medicine University of Mississippi Medical Center Jackson MS USA; ^5^ Department of Epidemiology Brown University School of Public Health Providence RI USA; ^6^ Wesleyan University Middletown CT USA; ^7^ The Fenway Institute Boston MA USA; ^8^ Division of Infectious Diseases Beth Israel Deaconess Medical Center Boston MA USA; ^9^ Department of Medicine Harvard Medical School Boston MA USA

**Keywords:** HIV prevention, PrEP, clinical care, adherence, retention in care

## Abstract

**Introduction:**

Retention in HIV pre‐exposure prophylaxis (PrEP) care in real‐world settings, outside of controlled trials or demonstration projects, remains poorly understood.

**Methods:**

We evaluated retention in PrEP care outcomes among men who have sex with men (MSM) and transgender women prescribed PrEP through March 2017 at three clinical sites in the United States (US): Jackson, Mississippi; Providence, Rhode Island; and St. Louis, Missouri. We determined retention rates by attendance of clinical visits every three months, per US Centers for Disease Control and Prevention (CDC) guidelines, as well as by the timing of patients’ actual clinical visits. Multivariable analyses examined demographic and behavioural factors associated with retention.

**Results:**

From 2013 to 2015, 282 MSM and transgender women were prescribed PrEP; 82% attended a follow‐up visit. Based on CDC recommendations, 56% of patients were retained in PrEP care at the first follow‐up visit, having attended a visit three months after initiation. However, 76% had a follow‐up visit within eight months. Thirty‐percent were retained at 12 months by CDC criteria, but 62% were retained when using a 16‐month endpoint. Self‐reported adherence was strongly correlated with retention. In multivariable analyses, younger age was associated with decreased odds of retention at initial follow‐up, and completing college was associated with increased odds of retention at 16 months. Eight participants were newly diagnosed with HIV; six were African American, and seven were under 30 years of age.

**Conclusions:**

Measuring retention in PrEP care using three‐month follow‐up intervals may underestimate true retention. Nevertheless, retention in PrEP care is suboptimal in real‐world settings and should be the focus of future interventions.

## 1 Introduction

The HIV epidemic in the United States (US) disproportionately impacts gay, bisexual and other men who have sex with men (MSM), as well as transgender women. MSM have a one in 11 lifetime risk of HIV acquisition in the US. This increases to one in five and one in two for Hispanic/Latino and African American MSM, respectively 1. Transgender women are also at increased risk with an HIV prevalence of up to 22% in the US 2. Pre‐exposure prophylaxis (PrEP) is a once‐daily, oral antiretroviral medication (co‐formulated tenofovir disoproxil fumarate and emtricitabine, TDF/FTC) with demonstrated efficacy in reducing HIV transmission among MSM and transgender women 3. Although PrEP programmes have expanded across the US 4, 5, 6 with over 100,000 individuals prescribed the medication 7, this is only a fraction of the 1,232,000 individuals the US Centers for Disease Control and Prevention (CDC) estimates are clinically indicated for PrEP 8. Many populations at highest risk for HIV acquisition do not have adequate access to PrEP. Rates of PrEP awareness, uptake and adherence have been lower among younger MSM and African Americans compared to white populations 6.

New frameworks outline opportunities for evaluating patient progression through the continuum of PrEP care. These frameworks measure public health outcomes related to PrEP awareness, linkage to care, initiation, adherence and retention in PrEP care 9. Despite the importance of retention in PrEP care to reduce HIV acquisition, little is currently understood about rates of retention outside of research settings and how demographic, social and structural factors influence retention. Our previous three‐site study in Rhode Island, Mississippi and Missouri found that 73% of patients prescribed PrEP were retained in care at three months and 60% were retained at six months 10. However, longer term outcomes of PrEP implementation efforts are not yet well understood. Structural, social, behavioural and clinical factors may undermine retention in PrEP care. Barriers may include medication and monitoring costs 11, access to care 12, medical mistrust 13, stigma related to sexual orientation 14, low self‐perceived HIV risk 15, lack of motivation 16 and limited provider knowledge and willingness to prescribe PrEP 17, as well as personal and community stigma 18 and promiscuity stereotypes 19 regarding PrEP users.

We evaluated initial and long‐term rates of retention in PrEP care at three clinical sites in Providence, Rhode Island; Jackson, Mississippi; and St. Louis, Missouri. We examined demographic and behavioural factors associated with retention in PrEP care using CDC guidelines and compared to actual patient visits for PrEP care at “real‐world” intervals. We also evaluated HIV seroconversions in this group of patients.

## 2 Methods

In 2013, PrEP implementation programmes were established in Providence, Rhode Island; Jackson, Mississippi; and St. Louis, Missouri. These programmes focused on delivering PrEP care in safety‐net and specialty care settings in three mid‐sized, diverse US cities including a community clinic (Mississippi) and infectious diseases specialty clinics (Missouri and Rhode Island). Individuals were typically provided a 30‐day supply of the medication to be taken daily with two to three refills in accordance with CDC recommendations, but this varied by provider and clinic site and was at times dictated by insurance companies 3. Refills could also be given for a longer duration based on provider discretion. Laboratory testing was generally conducted at each clinical visit per CDC recommendations which included creatinine (to assess renal function), HIV and other sexually transmitted infections (STIs) such as syphilis and oral/rectal/urogenital gonorrhoea and chlamydia. PrEP was offered to MSM and transgender women reporting condomless anal sex, individuals in HIV serodiscordant partnerships and other high‐risk populations, such as persons who inject drugs.

We reviewed clinical records for demographic, behavioural, clinical appointment history and laboratory data on MSM and transgender women prescribed TDF/FTC as PrEP through March 2017. Demographic information collected included age (<30 vs. ≥30 years old), gender (cisgender vs. transgender), race (African American vs. other race), ethnicity (Hispanic/Latino vs. not Hispanic/Latino), income (defined continuously, as well as dichotomously as < or ≥$15,000 per year), education (less than college education vs. college education or above) and insurance status. Behavioural data collected included number of sexual partners (≤5 vs. >5 sexual partners in the past three months), gender of sexual partners, condom use (any condomless anal sex in the past three months vs. none) and injection drug use. We also reviewed patient attendance at clinical visits. Adherence was defined as taking four or more daily pills in the previous seven days 20, according to patient self‐report at each clinical follow‐up visit. Self‐reported adherence has been shown to be a reliable indicator of true adherence 21. We also examined self‐reported seven‐day adherence defined as taking daily pills every day in the previous seven days.

The CDC recommends clinical follow‐up visits every three months 3. We evaluated initial retention in care based on CDC guidelines, which included attending an initial follow‐up visit at three months (±30 days), and long‐term retention in care as attending a follow‐up visit at 12 months (±30 days). In real‐world clinical settings, patients may not attend follow‐up appointments at precise three‐month intervals. Providers may also not be able to see patients at precise three‐month intervals. We therefore compared retention outcomes employing the CDC definition with actual patient follow‐up visit dates. We measured real‐world retention by evaluating patients who *ever* followed up and who reported PrEP adherence of four or more pills per week. Using a similar approach, we also evaluated long‐term retention in care rates.

Baseline and follow‐up characteristics for the study sample were described with means and standard deviations for continuous variables and proportions for categorical variables for the overall sample as well as by site. We used the chi‐square and Fisher's exact tests for categorical variables and the Kruskal–Wallis test for continuous variables to test for differences in characteristics across sites. We evaluated associations between self‐reported adherence and the number of days from initial PrEP appointments to the first follow‐up visits. To assess variables associated with retention in PrEP care, we built a series of log binomial regression models to determine prevalence ratios (PRs). For each dependent variable, a bivariate log binomial regression model was first built for each independent variable. Second, multivariable log binomial models, including all independent sociodemographic variables, were constructed for each dependent variable. The multivariable log binomial model for long‐term retention also included covariates of initial follow‐up within 120 days and seven‐day adherence at initial follow‐up. Secondary analyses examined retention by age and race within and between sites. All analyses were conducted in R 3.3.5 (R Foundation for Statistical Computing, Vienna, Austria).

All participants provided written informed consent to review of clinical data. The study protocol was approved by institutional review boards at each study site.

## 3 Results

Among 317 individuals prescribed PrEP during the study period, 85% were MSM, 8% were women who had sex with men, 4% were men who have sex with both men and women (MSM/F) and 3% were men who had sex with women. The primary analysis was restricted to transgender women (n = 2), persons reporting their gender as “other” (n = 1) and cisgender MSM (n = 279, including MSM/F; analysis total n = 282), of whom 54% were between the ages of 15‐30 years, 29% were African American and 11% were Hispanic/Latino. Twenty‐nine percent of participants reported having an HIV‐positive partner, and 23% reported having more than five male sexual partners in the preceding three months. Among MSM (n = 279), the majority (74%) reported condomless anal sex in the preceding three months (Table 1).

**Table 1:** Baseline characteristics of individuals prescribed HIV pre‐exposure prophylaxis in Providence, Rhode Island; Jackson, Mississippi; and St. Louis, Missouri

	Total (n = 282)		Rhode Island (n = 129)		Mississippi (n = 86)		Missouri (n = 67)		*p*‐Value^a^
	n	%	n	%	n	%	n	%	
Age									0.014
<30 years	151	53.5	58	45.0	56	65.1	37	55.2	
≥30 years	131	46.5	71	55.0	30	34.9	30	44.8	
Gender									
Cisgender man	279	98.9	128	99.2	85	98.8	66	98.5	1.00
Transgender woman	2	0.7	1	0.8	1	1.2	0	0.0	
Other	1	0.4	0	0.0	0	0.0	1	1.5	
Race (n = 281)									<0.001
White	160	56.9	93	72.7	24	27.9	43	64.2	
African American	81	28.8	6	4.7	58	67.4	17	25.4	
Asian	8	2.8	4	3.1	2	2.3	2	3.0	
Other	32	11.4	25	19.5	2	2.3	5	7.5	
Ethnicity									<0.001
Hispanic/Latino	30	10.7	26	20.3	2	2.3	2	3.0	
Education (n = 279)									0.026
Elementary	6	2.2	3	2.3	3	3.5	0	0.0	
High school	85	30.5	39	30.5	24	28.2	22	33.3	
College	133	47.7	60	46.9	49	57.6	24	36.4	
Graduate	55	19.7	26	20.3	9	10.6	20	30.3	
Insurance									
Private	197	69.9	99	76.7	42	48.8	56	83.6	<0.001
Public	34	12.1	26	20.2	4	4.7	4	6.0	
None	47	16.7	1	0.8	40	46.5	6	9.0	
Other	4	1.4	3	2.3	0	0.0	1	1.5	
Annual income, median (interquartile range)	27,000 (40,000)		40,000 (56,000)		13,000 (33,000)		27,000 (28,600)		<0.001
Annual income < $15K/year	92	33.7	32	25.2	43	53.1	17	26.2	<0.001
Gender(s) of sex partners^b^									
Men only	269	95.4	125	96.9	83	96.5	61	91.0	0.183
Men and women	13	4.6	4	3.1	3	3.5	6	9.0	
HIV‐positive male partner^b^	76	28.7	31	26.7	24	29.3	21	31.3	0.793
>5 male sex partners^b^	65	23.3	33	25.6	13	15.5	19	28.8	0.113
Condomless anal sex^b^	195	73.6	95	81.9	52	63.4	48	71.6	0.013
Lifetime injection drug use	12	4.3	10	7.8	0	0.00	2	3.0	0.010

^a^ *p*‐values were calculated using chi‐square tests; Fisher's exact test was used when expected cell counts were < 5; ^b^behaviour in the three months prior to pre‐exposure prophylaxis initiation.

**Figure 1:**
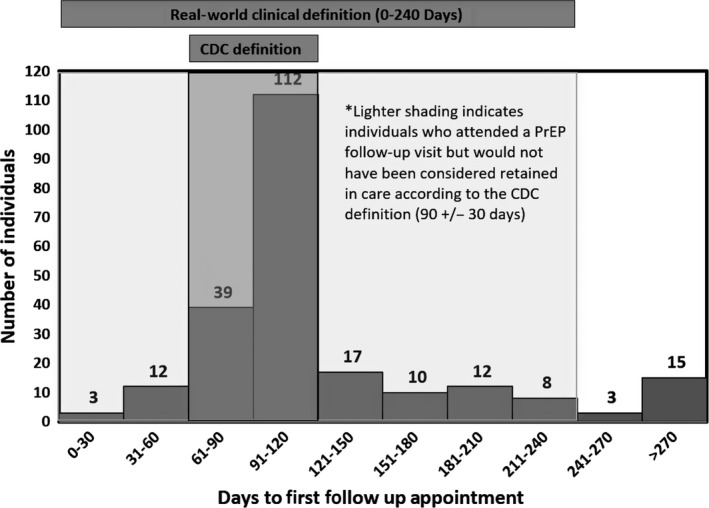
Distribution of the number of days from baseline to first follow‐up visit among individuals prescribed PrEP. CDC, Centers for Disease Control and Prevention; PrEP, pre‐exposure prophylaxis.

**Figure 2:**
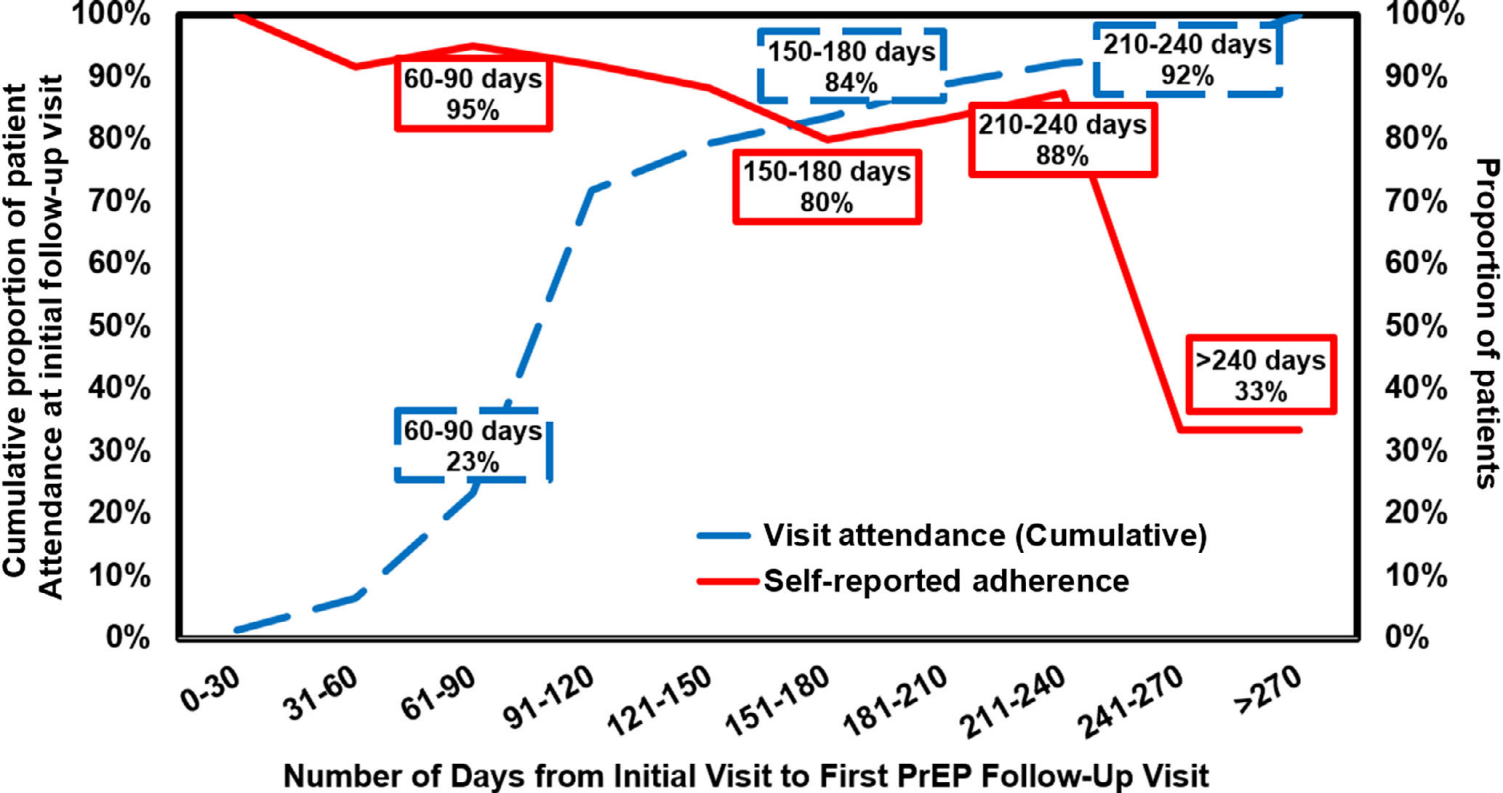
Relationship between retention in PrEP care and adherence. Retention in care was defined as the cumulative proportion of patient attendance at a first follow‐up visit over number of days since initial visit. Adherence was defined as the proportion of patients reporting ≥ 4 PrEP doses taken per week; 7.8% (N = 18/231) of patients had an initial PrEP follow‐up visit at > 240 days (eight months). PrEP, pre‐exposure prophylaxis.

The number of days between the initial PrEP appointment and first follow‐up appointment varied widely (Figure 1). The median number of days to initial follow‐up was 98. When measuring retention based on CDC guidelines, 54% (n = 151) of the total number of individuals (n = 282) followed up for an initial clinical visit at three months (±30 days). However, 76% (n = 213) of the total sample (n = 282) presented for an initial follow‐up visit within eight months, and 79% presented within 12 months. Among individuals who attended *any* follow‐up appointment (n = 231, 82% of the total sample), only 72% presented for care within four months; the remaining 28% followed up at a later time. Ninety‐two percent (n = 213) of individuals who followed up at any point during the study period (n = 231) did so within eight months. Among individuals who attended a PrEP follow‐up visit by eight months, self‐reported adherence was 88%. Self‐reported adherence dropped markedly among individuals whose first follow‐up appointment took place after eight months to 33% (Figure 2). Given this, even though the definition of retention was based on PrEP adherence, a minority of individuals were still considered retained but non‐adherent. Compared to patients who did not follow‐up, patients who had at least one follow‐up visit were more likely to be over 30 years of age, more educated and more likely to have private insurance.

**Figure 3:**
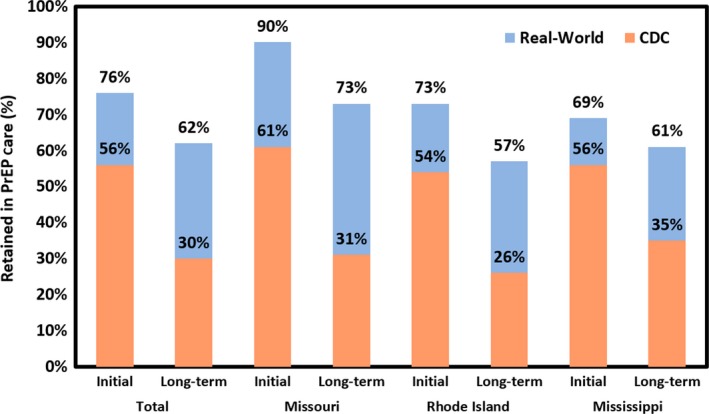
Retention in care among individuals prescribed PrEP across three clinical PrEP implementation programmes. Total and site‐specific retention in PrEP care among individuals attending clinical pre‐exposure prophylaxis (PrEP) implementation programmes in Mississippi (n = 86), Missouri (n = 67) and Rhode Island (n = 129; total n = 282). Initial retention was defined based on the Centers for Disease Control and Prevention (CDC) definition (three‐months ± 30 days) or a real‐world definition (by eight‐months). Long‐term retention was defined based on the CDC definition (12‐months ± 30 days) or a real‐world definition (8‐16 months).

We initially used the current CDC definition of retention in PrEP care but found this significantly underrepresented those who actually followed‐up. Therefore, we used the actual timing of patient visits to define retention in PrEP care. Using this cutoff of eight months based on real‐world presentation for initial retention in PrEP care, 76% of individuals (n = 213) were retained in care at their first visit, including 73% in Rhode Island, 69% in Mississippi and 90% in Missouri (*p* = 0.007, Figure 3). In unadjusted analyses, initial retention in care was significantly higher in Missouri than Mississippi (PR = 1.31; 95% CI: 1.11, 1.54; Table 2); this difference was no longer significant after adjusting for geographic and demographic characteristics (adjusted PR (aPR) = 1.10; 95% CI: 0.94, 1.05).

**Table 2:** PRs for retention in pre‐exposure prophylaxis care at initial and one‐year follow‐up visits, unadjusted and adjusted for geographic and demographic characteristics

	Initial follow‐up		One‐year follow‐up	
	PR (95% CI)	aPR (95% CI)	PR (95% CI)	aPR (95% CI)
Study site				
Mississippi	1.00 (ref)	1.00 (ref)	1.00 (ref)	1.00 (ref)
Missouri	1.31 (1.11, 1.54)^a^	1.10 (0.94, 1.28)	1.21 (0.97, 1.51)	0.97 (0.82, 1.14)
Rhode Island	1.06 (0.89, 1.27)	0.88 (0.74, 1.05)	0.95 (0.76, 1.19)	0.88 (0.66, 0.98)^a^
Age < 30 years	0.84 (0.74, 0.96)^a^	0.94 (0.84, 1.05)	0.88 (0.73, 1.05)	1.02 (0.89, 1.18)
African American/Black (ref: non‐AA/Black)	0.80 (0.67, 0.95)^a^	0.92 (0.76, 1.10)	0.89 (0.71, 1.10)	0.95 (0.79, 1.15)
Hispanic/Latino (ref: non‐Hispanic/Latino)	1.06 (0.88, 1.29)	1.12 (1.00, 1.25)^a^	1.09 (0.83, 1.43)	1.04 (0.83, 1.31)
College education or above (ref: less than college)	1.14 (0.97,1.33)	1.06 (0.92, 1.21)	1.34 (1.07, 1.68)^a^	1.11 (0.93, 1.33)
Income <$15,000 (ref: ≥$15,000)	0.89 (0.77, 1.04)	1.03 (0.91, 1.15)	0.86 (0.70, 1.06)	0.93 (0.79, 1.10)
No health insurance (ref: health insurance)	0.75 (0.59, 0.96)^a^	0.78 (0.59, 1.02)	0.75 (0.55, 1.02)	0.87 (0.63, 1.20)
Adherence to PrEP 4+ days/week at initial follow‐up visit			1.34 (0.95, 1.89)	
Adherence to PrEP seven days/week at initial follow‐up visit			1.18 (0.96, 1.45)	1.02 (0.84, 1.23)
Initial follow‐up visit within 120 days			1.90 (1.50, 2.41)^a^	1.13 (0.92, 1.37)

PR, prevalence ratio; aPR, adjusted PR; CI, confidence interval; AA, African American; ref, referent group; PrEP, pre‐exposure prophylaxis.

a *p *<* *0.05.

When employing retention measures based strictly on CDC guidelines, 30% of all patients (n = 282) followed up for long‐term clinical visits within 12 months (±30 days). Given that individuals prescribed PrEP initially presented within a range of eight months, we defined long‐term retention as attending a single visit within an eight month window from 8 to 16 months. When using this cutoff based on patients’ real‐world presentation for long‐term follow‐up visits, 62% of patients presented within 16 months and were considered retained in PrEP care (Figure 1). A total of 57% of MSM were retained in PrEP care in Rhode Island, 61% were retained in Mississippi and 73% were retained in Missouri. Self‐reported adherence for four or more days in the week prior to the one‐year follow‐up visit was 83% and seven‐day adherence was 74%. There were no significant differences in retention in PrEP care between sites at the long‐term endpoint (*p* = 0.091; Figure 3, Table 2). Retention in care rates using our real‐world definition at 8 and 16 months were significantly higher when compared to retention in care rates using the CDC guidelines for 3 and 12 month follow‐up, both by site and overall (*p* < 0.001).

Based on these trends, which reflect patients’ actual presentation for services, the multivariable analyses employed the definition for initial retention in PrEP care as attending a follow‐up visit within eight months of PrEP initiation. Similarly, we defined long‐term retention in PrEP care as attending a follow‐up visit from 9 to 16 months after PrEP initiation.

In the bivariate analyses, being under 30 years of age was significantly associated with decreased prevalence of initial retention in PrEP care (PR = 0.84; 95% CI: 0.74, 0.96) compared to age 30 years and over, but not with retention at one year (Table 2). African American individuals had significantly reduced prevalence of being retained in PrEP care at the initial time point (PR = 0.80; 95% CI: 0.67, 0.95) compared to individuals of any other race, but not at one year. Uninsured patients had significantly reduced prevalence of being retained at the initial time point (PR = 0.75; 95% CI: 0.59, 0.96) compared to those with health insurance (Table 2). College education was associated with increased prevalence of retention at one year in bivariate analyses (PR = 1.34; 95% CI: 1.07, 1.68). Adherence to PrEP medication for four or more days at initial follow‐up was not associated with increased prevalence of retention at one year in bivariate analyses, nor was seven day adherence (Table 2). Finally, having an initial follow‐up visit within four months was associated with increased prevalence of one‐year retention in bivariate analyses (PR = 1.90, 95% CI: 1.50, 2.41). In the multivariable analysis, Hispanic individuals had significantly greater prevalence of initial retention compared to non‐Hispanic individuals (aPR = 1.12; 95% CI: 1.00, 1.25). Rhode Island had significantly lower prevalence of retention compared to Mississippi (aPR = 0.88; 95% CI: 0.66, 0.98) at one year (Table 2).

Additional analyses examining retention by age and race within each site at eight and 16‐month endpoints indicated that Mississippi individuals had significantly lower prevalence of retention at eight months if under the age of 30, but the difference was not significant at 16 months. In Mississippi, 57% of patients under the age of 30 were initially retained, compared to 90% of patients age 30 or older (*p* = 0.001). There were no significant differences by age at either time point for Rhode Island and Missouri. Mississippi retained a lower prevalence of African American patients at the initial and long‐term endpoints. At initial follow‐up, 57% of African American individuals in Mississippi were retained in care, compared to 93% of others (*p* < 0.001). At long‐term follow‐up, 53% of African American individuals were retained compared to 75% of non‐African Americans (*p* = 0.039). There were no significant race differences in initial and one‐year retention endpoints in Rhode Island or Missouri.

We also compared patients who had a follow‐up visit within four months of an initial visit to patients who had a follow‐up visit within 5‐8 months of an initial visit on likelihood of an increase in creatinine between initial visit and follow‐up and likelihood of having a new positive STI test between initial visit and follow‐up. There were no significant differences between groups in likelihood of creatinine increase or new STI. Of the 213 patients who had a follow‐up visit, a total of 58 (52.7%) patients who had a follow‐up visit within 1‐4 months showed an increase in creatinine at follow‐up compared to n = 15 (46.9%) of patients who followed up within 5‐8 months (*p* = 0.560). A total of 22 (13.3%) of patients who followed up within 1‐4 months tested positive for a new STI at follow‐up compared to n = 6 (12.8%) of patients who followed up within 5‐8 months (*p* = 0.930).

Three percent (n = 8) of individuals prescribed PrEP were newly diagnosed with HIV during the course of the study period (Mississippi: n = 4; Rhode Island: n = 2; Missouri: n = 2). The approximate HIV incidence was 1.14 per 100 person years. Six individuals (75%) who were newly diagnosed were African American, all but one were younger than 30 years old, four (50%) were college educated and four (50%) had an income of <$15,000 per year. All African American patients were younger than 30 years old. Among the eight individuals who were newly HIV diagnosed, four individuals tested positive for HIV at baseline before initiating PrEP and three reported suboptimal adherence. One individual seroconverted at the first follow‐up appointment, suggesting acute HIV infection upon presentation or seroconversion caused by suboptimal medication adherence.

## 4 Discussion

This is among the first studies to evaluate longer term rates of retention in PrEP care at real‐world clinical programmes in the US. We found that individuals prescribed PrEP in settings outside of research presented for care at irregular intervals. Nearly one‐third of individuals presented for follow‐up after four months but were still retained in PrEP care. This can be attributed to several potential reasons. Patients may not present regularly for care, particularly if they are socially disadvantaged, generally healthy and not accustomed to seeking medical care prior to PrEP initiation. Providers may also not be available to see patients at precise three‐month intervals. Otherwise healthy patients may not always prioritize preventive health visits or experience other structural challenges that may delay their presentation to care. We have previously described the study settings and evaluated structural barriers to care that exist at our clinics that specifically highlight insurance‐related and financial challenges 11, 22, 23. Importantly, providers may also fill prescriptions without a patient presenting for care at three‐month intervals, particularly if they believe the short‐term benefits of refilling PrEP prescriptions outweigh HIV acquisition risks. Although there was no formal policy, this was noted to commonly occur across the study sites. Future studies should explore the risks versus benefits of prescribing PrEP past the standard three‐month interval.

Retention in care and self‐reported adherence were highly correlated up until approximately eight months after PrEP initiation. Individuals who returned for their first follow‐up visit within eight months of their initial appointment reported high levels of adherence to PrEP. Adherence was significantly lower in individuals who attended their first follow‐up visit after eight months. This eight‐month drop off is attributed to providers prescribing refills without patients presenting at regular intervals, or even presenting at all, during the early follow‐up period. These phenomena prompted us to develop alternate metrics to more rigorously examine retention in PrEP care. Given that the definition of retention is often defined on more stringent CDC‐based criteria, the approach used in this study allowed an objective measurement of retention in PrEP care based on actual patient visits. This definition was notably more liberal than using a CDC‐based definition and was more inclusive of individuals who actually followed up. This study offers an alternative approach to evaluate retention in PrEP care based on real‐world outcomes. Future studies should evaluate this definition compared to other potential approaches such as those used for retention in HIV care (e.g. the number of clinical visits in a defined timeframe).

Long‐term retention in PrEP care was similar across all sites using the alternate, real‐world metric. Using this metric, retention rates declined between initial and long‐term follow‐up (76% and 62% retention respectively). At initial follow‐up as defined by the real‐world metric, a significantly lower prevalence of individuals under 30 years of age were retained in PrEP care compared to older individuals. A significantly greater prevalence of Hispanic individuals were retained in PrEP care in after adjusting for clinical site and demographic factors. Race was not significant after adjusting for clinical site and demographic factors. Finally, three percent of HIV‐negative individuals who were prescribed PrEP at these programmes seroconverted, the majority of whom were young African American MSM. The approximate HIV incidence rate was 1.14 per 100 person years.

Notably, younger age was a significant predictor of not being retained in PrEP care at the initial visit, particularly in Mississippi, which had the greatest age discrepancy. This is an unsurprising finding, given that younger age has also been associated with significantly lower PrEP awareness 24, interest 25, uptake 12 and adherence 26. Similarly, among individuals living with HIV, younger age is also significantly associated with lower retention in care 27. These findings are similar to those observed in the Adolescent Medicine Trials Network 110 study, an open‐label PrEP demonstration project which enrolled young MSM across 12 US cities 28.

Earlier studies suggest that African Americans may be less likely than other groups to be retained in PrEP care at six‐month time points 4, 10. We found that African American individuals in Mississippi had a significantly lower prevalence of retention in care compared to white individuals at initial and long‐term follow‐up visits. Previous research has found significant racial disparities in PrEP uptake and other PrEP care continuum endpoints 29, 30, as well as across the HIV care continuum 31, 32. Racial differences in retention in care were observed in our previous study among a similar population, where the follow‐up period was attenuated at the six‐month endpoint 10. Other studies have found lower PrEP awareness and uptake among African American and Hispanic/Latino populations compared to white populations 33, 34. Our study was underpowered to detect differences among racial and ethnic groups, particularly when stratified by clinical site. However, these preliminary trends underscore the need for ongoing research examining racial and ethnic disparities across the PrEP continuum.

Three percent of individuals were newly diagnosed with HIV during the study, suggesting that real‐world PrEP programmes are engaging populations that are at risk for HIV. However, the high incidence and suboptimal retention rates underscore the need for further efforts to identify more effective strategies to promote adherence and retention in PrEP care, especially for African Americans. Our HIV diagnosis rate suggests adherence and retention in care may be suboptimal among individuals prescribed PrEP in other US settings. The majority of patients (75%) who were newly HIV diagnosed in this study were young African American MSM. Furthermore, the three percent of individuals who were diagnosed may underrepresent the true number of seroconversions, given that individuals not retained in care may have even higher seroconversion rates.

These study findings are subject to several limitations. First, our sample size may have been underpowered to detect associations between race or ethnicity and retention in PrEP care. Given an emerging body of evidence highlighting racial and ethnic disparities across the PrEP care continuum 33, 35, 36, larger samples may help elucidate differences in retention in care by race and ethnicity. Further study is also needed among transgender populations to determine PrEP outcomes. Given the real‐world nature of our study, we relied on self‐reported adherence. Early clinical efficacy trials demonstrated low TDF/FTC blood concentrations despite high self‐reported adherence 37. However, PrEP programmes in real‐world settings have found adherence is high among those retained in PrEP care and that self‐reported adherence is a good proxy for TDF/FTC blood levels 38. In this study, TDF/FTC blood levels and prescription refill data were unavailable. Intermittent PrEP use could also affect observed adherence and retention rates. PrEP is not approved for intermittent use in the US and was not prescribed in this manner but could have been possible among a subset of individuals. Importantly, variability in times between initial PrEP visits and subsequent follow‐up visits poses challenges for defining and evaluating retention outcomes.

Our findings suggest that using CDC guidelines to measure retention likely underestimates true retention in PrEP care in real‐world settings. Using CDC criteria, many individuals who were ultimately retained in care would have been considered lost to follow‐up. While the majority of patients presented within four months, approximately one‐third of those who ever presented for follow‐up care did so after four months. Our findings suggest there is substantial variability in timing of actual patient visits, and that PrEP programmes may need to develop strategies to prevent gaps in medication adherence when patients present at irregular intervals. These findings are likely generalizable to many other real‐world settings. Further research is needed to determine if these retention patterns have an impact on other outcomes (e.g. other STIs).

These findings nevertheless underscore suboptimal retention in PrEP care, and the need for wrap‐around services to promote adherence and retention in care among young and African American MSM. The Ryan White HIV/AIDS Program is the largest funder of HIV care and related wrap‐around services, including services to promote adherence and retention in HIV care 39. However, Ryan White services are currently reserved for individuals who have HIV and lack health insurance. Expanding Ryan White eligibility to individuals who are at high risk for HIV acquisition but are HIV‐negative could help address these critical challenges in retaining individuals in PrEP services.

## 5 Conclusions

Retaining individuals at highest risk for HIV transmission in PrEP services is paramount to maximize implementation and achieve the population health goal of reducing HIV incidence. Our results highlight the urgent need for culturally tailored, real‐world interventions to measure and to enhance retention in PrEP care, particularly for young African American MSM.

## Competing interests

The authors declare that they have no competing interests.

## Authors’ contributions

PAC, RP, LM, KM and AN conceived of the study. BDLM and JR developed the analytic plan and conducted formal statistical analysis. PAC, CSC, MCM and AN prepared the original manuscript draft; RRP, LM, BDLM, JT, CS and JR contributed to reviewing and editing the manuscript. All authors reviewed and approved the final manuscript.
